# The Effect of Hydrogen Bonding in Enhancing the Ionic Affinities of Immobilized Monoprotic Phosphate Ligands

**DOI:** 10.3390/ma10080968

**Published:** 2017-08-18

**Authors:** Spiro D. Alexandratos, Xiaoping Zhu

**Affiliations:** 1 Department of Chemistry, Hunter College of the City University of New York, New York, NY 10065, USA; zhuxiaoping2009@gmail.com; 2 Graduate Center of the City University of New York, New York, NY 10016, USA

**Keywords:** polymer, ligand, metal ions, separations

## Abstract

Environmental remediation requires ion-selective polymers that operate under a wide range of solution conditions. In one example, removal of trivalent and divalent metal ions from waste streams resulting from mining operations before they enter the environment requires treatment at acidic pH. The monoethyl ester phosphate ligands developed in this report operate from acidic solutions. They have been prepared on polystyrene-bound ethylene glycol, glycerol, and pentaerythritol, and it is found that intra-ligand hydrogen bonding affects their metal ion affinities. The affinity for a set of trivalent (Fe(III), Al(III), La(III), and Lu(III)) and divalent (Pb(II), Cd(II), Cu(II), and Zn(II)) ions is greater than that of corresponding neutral diethyl esters and phosphonic acid. In an earlier study, hydrogen bonding was found important in determining the metal ion affinities of immobilized phosphorylated polyol diethyl ester coordinating ligands; their Fourier transform infrared (FTIR) band shifts indicated that the basicity of the phosphoryl oxygen increased by hydrogen bonding to auxiliary –OH groups on the neighboring polyol. The same mechanism is operative with the monoprotic resins along with hydrogen bonding to the P–OH acid site. This is reflected in the FTIR spectra: the neutral phosphate diethyl ester resins have the P=O band at 1265 cm^−1^ while the monoethyl ester resins have the band shifted to 1230 cm^−1^; hydrogen bonding is further indicated by the broadness of this region down to 900 cm^−1^. The monoprotic pentaerythritol has the highest metal ion affinities of the polymers studied.

## 1. Introduction

The complexation of metal ions by polymer-supported reagents is important to many applications including recovery in environmental remediation [1], pre-concentration in analytical determinations [2], and pharmaceuticals [3]. The extent of complexation is determined by the stability of the complex. Depending on the ligand, complexation of metal ions can involve ion exchange or coordination. Metal ions can coordinate to neutral oxygen such as the phosphoryl oxygen [4] or nitrogen donors such as the pyridyl nitrogen [5], or bond ionically with ion exchange ligands such as carboxylate [6]. Differences in the strength of the coordinate covalent bond have been used to separate trivalent actinides from lanthanides [7]. More than one ligand can coordinate to metal ions and this is the basis of synergistic extraction [8].

Phosphorus-based substituents form a versatile set of ligands which permit the synthesis of complexants with a wide range of metal ion affinities [9]. Immobilized phosphinic, phosphonic, and phosphoric acids are important ion-complexing reagents because of their ability to ion exchange with metal ions under dilute acid conditions and coordinate to metal salts through the phosphoryl oxygen under more acidic solutions. Hydrogen bonding has been found to increase the ionic affinities of neutral coordinating polymers: diethyl phosphate ligands immobilized onto polystyrene through polyol scaffolds have increased ionic affinities due to hydrogen bonding at the phosphoryl oxygen by the –OH groups of the polyol [10]. The affinity of the phosphoryl oxygen for divalent ions increased as their polarizability increased and was significant from 0.01 M HNO_3_, but greatly decreased as the solution acidity increased.

**Figure 1:**
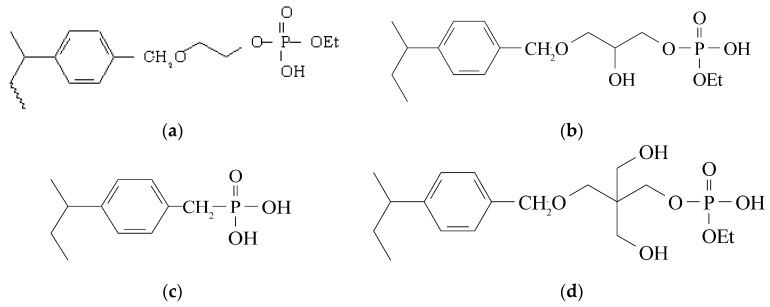
(**a**) Monoprotic phosphorylated ethylene glycol (pEG1M); (**b**) glycerol (pGlyM); (**c**) diprotic phosphonic acid (DPA); (**d**) pentaerythritol (pPentaM).

Having established the importance of hydrogen bonding between the phosphoryl oxygen and an –OH group on a neighboring polyol, the focus of subsequent study became the effect of hydrogen bonding between the phosphoryl oxygen and an acidic proton in the presence of a neighboring polyol from glycerol (Gly) and pentaerythritol (Penta), in the absence of a neighboring –OH using ethylene glycol (EG1) as a spacer from the polystyrene, and with only the acidic –OH in diprotic phosphonic acid (DPA). Figure 1 shows the structures of the monoprotic phosphorylated ethylene glycol (pEG1M), glycerol (pGlyM), and pentaerythritol (pPentaM), as well as DPA. A recent report has shown an effect of hydrogen bonding in the affinity for Eu^3+^: monoprotic glycols have higher affinities compared to DPA due to decreased hydrogen bonding [11]. Given that the affinities for divalent transition metal ions by the purely coordinating diethyl ester resins decreased to negligible levels as acidity increased from 0.01 M HNO_3_ [10], the objective of the current report was to determine whether divalent and trivalent metal ion affinities of phosphate ligands capable of ion exchange would be affected by hydrogen bonding and whether these polymers would have higher affinities than neutral coordinating phosphate ligands. These polymers are analogous to the neutral phosphates but with one –OEt group replaced by–OH. The metal sorption behavior for a set of divalent (Pb(II), Cd(II), Cu(II), and Zn(II)) and trivalent (Fe(III), Al(III), La(III), and Lu(III)) ions by the monoprotic polymers was compared to the coordinating polymers as well as the phosphonic acid. The metal ions were chosen as representative of a range of polarizabilities [12].

## 2. Experimental

Copolymers of vinylbenzyl chloride (VBC) and divinylbenzene (DVB) were prepared by suspension polymerization with 0.5 wt % benzoyl peroxide as initiator. The polyVBC beads were microporous and crosslinked with 2% DVB. They were extracted with toluene, dried, and sieved to retain a 250–420 µm particle size. All chemicals (VBC, DVB, polyols, diethyl chlorophosphate, organic solvents, metal nitrate salts, and metal standard solutions) were purchased from Fisher Scientific (Waltham, MA, USA).

### 2.1. Synthesis

#### 2.1.1. Synthesis of Diprotic Phosphonic Acid (DPA) Polymer

Copolymer beads (10.0 g) were refluxed with 100 mL of triethyl phosphate for 17 h. The beads were washed with acetone, aqueous acetone, water, and refluxed with 50 mL of concentrated HCl for 17 h. After washing with water, the beads were conditioned with 1 L of each of 1 M NaOH, H_2_O, 1 M HCl, and H_2_O.

#### 2.1.2. Synthesis of Phosphorylated Ethylene Glycol Monoprotic (pEG1M) Polymer

Ten grams of NaH (60% dispersion) were added to 100 g of ethylene glycol and 100 mL of dioxane in a 250 mL round-bottom flask fitted with a condenser, stirrer, and gas inlet tube. The mixture was stirred at 23 °C under N_2_ for 2 h, then 6.0 g of copolymer beads swollen in 40 mL of dioxane for 2 h were added. The reaction was refluxed for 17 h. The solution was removed, the beads were washed with dioxane, dioxane/water (1:1) and water, and then vacuum-dried at 70 °C for 12 h. The beads (2.0 g) were phosphorylated by stirring in 100 mL of pyridine and 10.0 g 4-dimethylamino-pyridine (DMAP) for 2 h, then adding 10 mL of diethyl chlorophosphate (DECP), refluxing for 17 h, washing with 100 mL each of methanol, methanol/water (1:1), and water, and conditioning as above.

#### 2.1.3. Synthesis of Phosphorylated Glycerol Monoprotic (pGlyM) Polymer

Eight grams of NaH (60% dispersion) was added to 60.0 g of glycerol and 250 mL of *N*-methylpyrrolidone (NMP) in a 500 mL flask fitted with a condenser, stirrer, gas inlet tube, and thermometer. The mixture was stirred at 23 °C under N_2_ flow for 2 h. Copolymer beads (6.0 g) swollen in 50 mL of NMP for 2 h were then added to the solution. The reaction was kept at 80 °C for 24 h. The solution was removed and the beads washed with 100 mL each of NMP, NMP/water (1:1), and water, then vacuum-dried at 70 °C for 12 h. Phosphorylation followed the procedure above in 100 mL pyridine and 10.0 g DMAP.

#### 2.1.4. Synthesis of Phosphorylated Pentaerythritol Monoprotic (pPentaM)

The procedure was as above except that 60.0 g pentaerythritol in 300 mL NMP was used.

### 2.2. Characterization

The polymer acid capacities were determined by stirring 0.50 g Buchner-dried beads with 50 mL of 0.1000 M NaOH containing 5% NaCl for 17 h and then titrating a 10 mL aliquot with 0.1000 M HCl. The percentage solids (i.e., the ratio of dry to wet bead weight) was determined by Buchner-drying the beads for 5 min to remove excess water and then oven-drying at 110 °C for 12 h. The phosphorus capacity was measured after mineralizing 20 mg of resin in concentrated sulfuric acid in the presence of copper sulfate and subsequent reaction with ammonium vanadate-molybdate [13]. The intensity of yellow coloration was measured at 470 nm on Spectronic 21D (Milton Roy, Warminster, PA, USA). FTIR spectra were recorded on a Bomem Fourier Transform Infrared Spectrometer (ABB Bomem, Quebec, QC, Canada).

### 2.3. Metal Ion Affinities

Affinities were quantified by batch equilibration of the polymers with 10^−4^ M metal ion solutions at varying concentrations of HNO_3_. All pH values are calculated as the −log of the HNO_3_ concentrations except for the 0.01 M HNO_3_ solution used for the divalent ions which was the only one whose value changed from the one calculated (the experimental value is shown). Enough Buchner-dried DPA, pEG1M, pGlyM, and pPentaM to give 0.5 mmol P were shaken with 5 mL of metal ion solution for 17 h after being pre-equilibrated with the appropriate background solutions. Metal ion concentrations were determined by inductively coupled plasma-atomic emission spectrometry (PerkinElmer Optima 7000 DV ICP-AES, PerkinElmer, Waltham, MA, USA). The percent sorption was calculated from metal ion concentrations before and after equilibration.

## 3. Results

A method was developed to immobilize the polyols onto the polyVBC support by reaction with sodium hydride. Excess ethylene glycol in dioxane provided the highest yield while immobilization of glycerol and pentaerythritol was done in NMP because of their low solubility in dioxane. Elemental analyses and FTIR spectra showed all –CH_2_Cl groups reacted.

**Figure 2:**
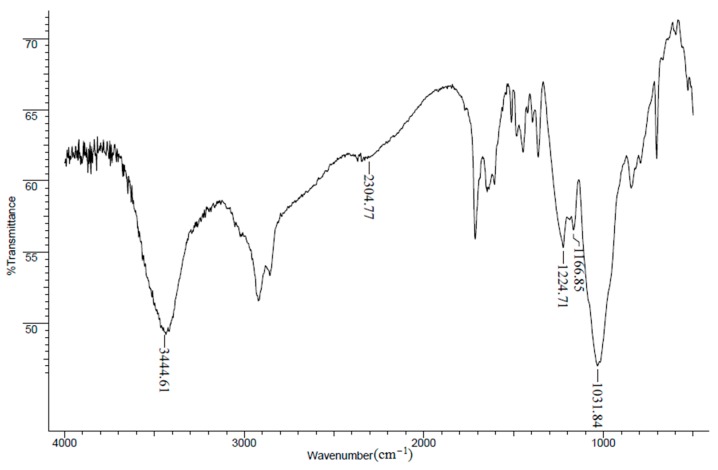
FTIR spectrum of pPentaM.

Reaction of polyols with DECP is known to give neutral phosphate esters [14]. The optimum procedure for directly preparing the monoprotic ligands was determined to be phosphorylating the polyols with DECP in pyridine containing 10% DMAP. Monoesters have been prepared with DMAP [15]. pEG1M, pGlyM, and pPenatM all have nearly equal acid and phosphorus capacities (Table 1), indicating that they consist of monoprotic phosphoric acid groups. As expected, DPA has an acid to phosphorus ratio of 2:1. The theoretical phosphorus capacities of monophosphorylated glycol, glycerol, and pentaerythritol are 3.36, 3.04, and 2.67 mmol/g, respectively; the experimental values for the latter two are within experimental error of theory, while the value for pEG1M is lower by 0.5 mmol/g. The FTIR spectrum of pPentaM is representative with the hydroxyl band at 3400 cm^−1^ and the acidic –OH at 2300 cm^−1^ (Figure 2). The P=O band appears at 1265 cm^−1^ in the diethyl phosphate and its conversion to the monoprotic pPentaM shifts the P=O band to 1224 cm^−1^. The significance of the shift will be discussed.

**Figure 3:**
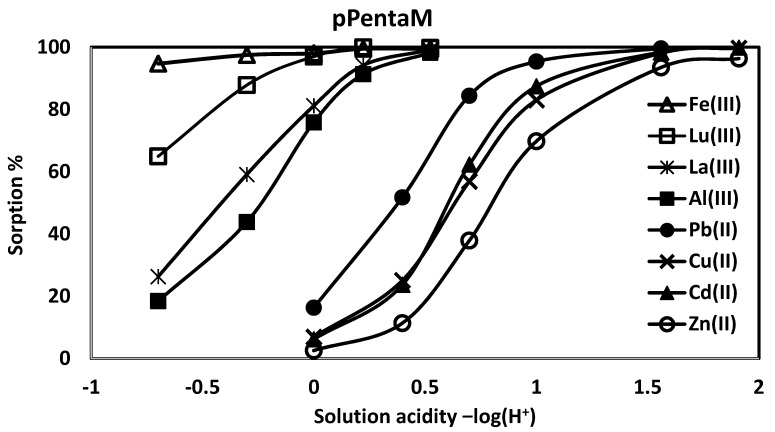
Divalent and trivalent metal ion affinities for pPentaM.

Table 2 shows the order of divalent ion affinities for the three monoprotic phosphates is Pb(II) > Cd(II) > Cu(II) > Zn(II), thus following the polarizability order set by the Misono softness parameter [16]. The order is the same as the diethyl esters, but with far higher affinities for all ions (the diester pEG1 had no affinity for the divalent ions even at pH 2) [10]. Though the three monoprotic phosphates operate through a common –OP(O)(OH)(OEt) ligand, they have different ionic affinities, with pPentaM > pGlyM > pEG1M; this order is maintained with the trivalent ions, with Fe(III) > Lu(III) > La(III) > Al(III). Figure 3 illustrates the results for pPentaM. Introducing an acidic P–OH group thus enhances significantly the metal ion affinity from acidic solutions compared to sorption by the neutral diethyl esters which have lower metal ion affinities due to H^+^ competition for the binding sites; e.g., though the neutral pGly has a high affinity for La(III) from 0.01 M nitric acid, it complexes only 11% from 1.0 M HNO_3_, while pGlyM complexes 90%.

The monoprotic ligands have greater affinities than diprotic phosphonic acid (Table 3); e.g., whereas DPA sorbs 52%, 25%, and 8% Lu(III) from 1, 2, and 5 M HNO_3_, pGlyM sorbs 92%, 74%, and 49% Lu(III) from the same solutions. Though fully functionalized polystyrene-bound phosphoric acid could not be prepared, the differences in affinity between phosphoric and phosphonic acids are not significant: the phosphoric acid ligand bound to poly(glycidyl methacrylate) has a low affinity for La(III) from a 1 M HNO_3_ solution and complexes 50% La(III) at pH 0.33 [17] while polystyrene-bound phosphonic acid has the same low La(III) affinity from 1 M HNO_3_ and complexes 50% of the La(III) at pH 0.63 [18]. Both have the same affinities for trivalent ions: Fe(III) >> Lu(III) > Al(III) > Gd(III) > La(III) > Cr(III).

**Figure 4:**
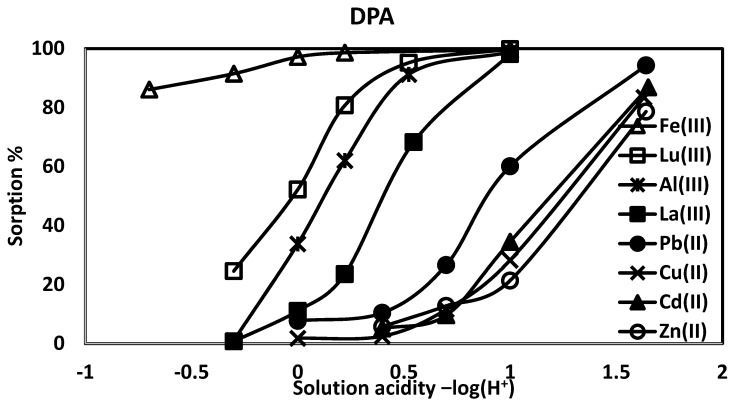
Divalent and trivalent metal ion affinities for the diprotic phosphonic acid, DPA.

The DPA affinities for divalent and trivalent ions are summarized in Figure 4. Over a range of solution acidities, DPA shows an order of Fe(III) > Lu(III) > Al(III) > La(III) > Pb(II) > Cd(II), Cu(II) > Zn(II); the monoprotic phosphates have nearly the same sequence: Fe(III) > Lu(III) > La (III) > Al(III) > Pb(II) > Cd(II), Cu(II) > Zn(II). There is a very high Fe(III) affinity which is less dependent on solution acidity, though at 5 M HNO_3_, more Fe(III) is sorbed by pPentaM (95%) than DPA (86%).

**Figure 5:**
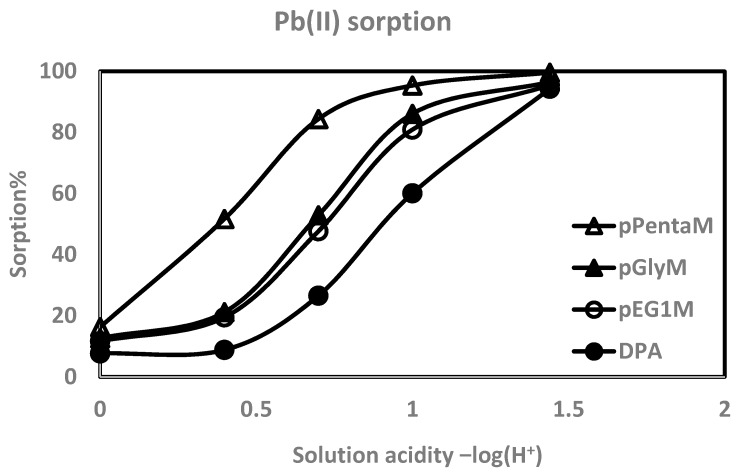

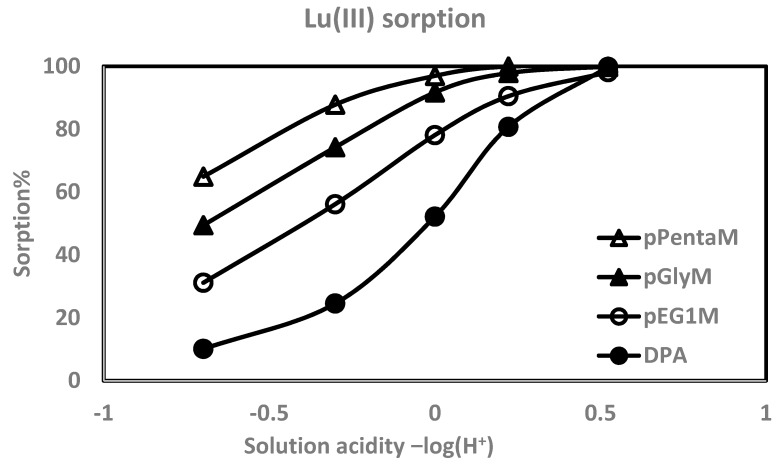
Metal ion affinities among the polymers: pPentaM > pGlyM > pEG1M > DPA.

The higher affinities of the monoprotic phosphates over the phosphonic acid are illustrated in Figure 5, with Pb(II) and Lu(III) as representative examples. There are differences among the monoprotic phosphates: pEG1M, with no –OH groups, has the lower affinities, sorbing 39% Al(III), 79% La(III), and 78% Lu(III), while pPentaM sorbs 76% Al(III), 81% La(III), and 97% Lu(III) from 1.0 M nitric acid. The same trend is found with divalent metal ions: from 0.1 M HNO_3_, pEG1M complexes 81% Pb(II), 55% Cd(II), 54% Cu(II), and 24% Zn(II), while pPentaM sorbs 95% Pb(II), 87% Cd(II), 83% Cu(II), and 70% Zn(II). Metal ion sorption is thus affected by the auxiliary hydroxyl groups, as it is with the diethyl ester phosphates; this will be discussed in the next section.

## 4. Discussion

**Figure 6:**
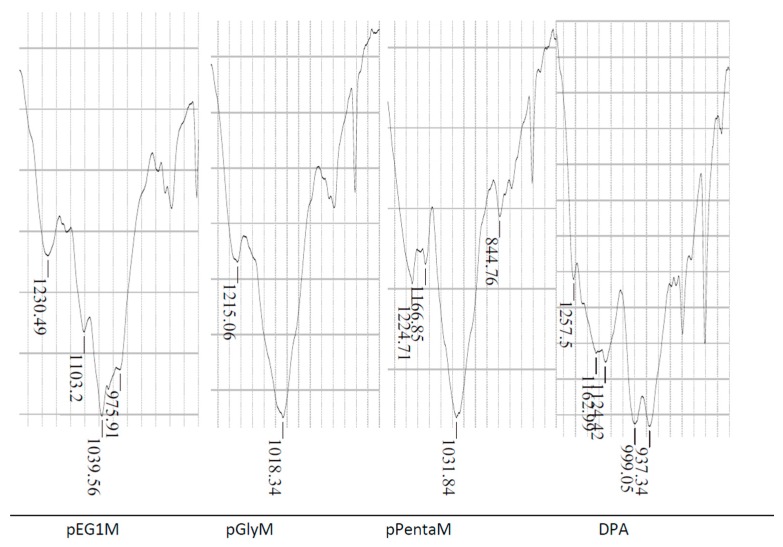
FTIR spectra of monoprotic (pEG1M, pGlyM, pPentaM) and diprotic (DPA).

Immobilized polyols can be phosphorylated with DECP in the presence of DMAP to form the monoprotic ligand. As noted earlier, the P=O band appears at 1265 cm^−1^ in the diethyl phosphate. FTIR spectra of the monoprotic phosphates show that replacing one –OEt group with an acidic –OH affects band broadness and positions between 900 and 1250 cm^−1^ (Figure 6); thus, for example, conversion to monoprotic pPentaM shifts the P=O from 1265 cm^−1^ to 1224 cm^−1^. The bands at 1124–1163 cm^−1^ in DPA are assigned to P=O and those at 999 and 937 cm^−1^ to the P–O(H). Though a phosphonate is being compared to phosphates, the additional downshift from two electron-withdrawing –OH groups is important to understanding the significance of the band positions when the acidic –OH group is proximate to the phosphoryl oxygen in the monoprotic phosphates (see below).

Electron withdrawing groups shift the P=O band to higher frequency as seen in the shifts of soluble phosphates and phosphine oxides: The P=O band for tributyl phosphine oxide is at 1155 cm^−1^ while that for triphenyl phosphine oxide is at 1190 cm^−1^, an increase of 35 cm^−1^ due to the electron withdrawing phenyl group [19]. The P=O in tributyl phosphate (TBP) is at 1282 cm^−1^, while that for triphenyl phosphate (TPP) is at 1296 cm^−1^, an increase of 14 cm^−1^ since the inductive effect is attenuated by the intervening oxygen atom.

Comparing phosphates to phosphine oxides shows the strong electron withdrawing effect of the –O– and its corresponding effect on P=O band positions. This affects the metal ion affinities of soluble complexants with phosphine oxides > phosphonates > phosphates [20]. Removal of one phenyl group from TPP to give the monoprotic (PhO)_2_P(O)(OH) lowers the P=O band by 110 cm^−1^ to 1187 cm^−1^ since an electron withdrawing group is removed (though the –O– remains bonded to the phosphorus). The decrease, however, is more intense than expected given the aforementioned attenuation. Moreover, removing one butyl group from TBP to give (BuO)_2_P(O)(OH) again results in a lowering of the P=O band position by 70 cm^−1^ to 1212 cm^−1^ despite removal of an electron donor. The –OH group must thus have an effect additional to its inductive effect since replacing it with the equally electron withdrawing –Cl to give (PhO)_2_P(O)Cl results in a shift to a much higher frequency (1308 cm^−1^).

It is proposed that the dominant effect of the acidic –OH in the phosphates which shifts the P=O band to lower frequencies is hydrogen bonding to the phosphoryl oxygen. Hydrogen bonding between amines to P=O has been reported [21]. This is now suggested to occur between the P=O and the acid site in immobilized phosphates: replacing –OEt on the phosphate diethyl ester with –OH shifts the P=O to lower frequency by 35 cm^−1^. Broadening around 1220 cm^−1^ has been attributed to hydrogen bonding at the phosphoryl oxygen [22] and the broad bands associated with the P=O (Figure 6) support the presence of intra- and intermolecular hydrogen bonding. This P–OH···O=P bonding in the monoprotic phosphates also decreases electron density in the phosphoryl bond, shifting the band from 1265 cm^−1^ in the neutral diethyl phosphate to 1215–1230 cm^−1^. Hydrogen bonding among soluble phosphonic acids has been observed [23,24]. This bonding is strong, as indicated by the fact that the phosphonic acid remains a dimer even after one proton is exchanged for a metal ion.

The metal ion affinities of pPentaM > pGlyM > pEG1M is the same as that for the corresponding neutral diesters (pPenta > pGly > pEG1). FTIR spectra show hydrogen bonding in the latter between its phosphoryl oxygen and the –OH groups on the neighboring glycol. The much increased affinity for metal ions by pEG1M relative to pEG1 is not only from the ion exchange component but also from the P=O/ acidic –OH hydrogen bonding which results in a lower P=O electron density, as indicated by the FTIR band shift, and corresponding increased Lewis basicity of the phosphoryl oxygen. That basicity is enhanced in pGlyM and pPentaM due to supplementary hydrogen bonding from the polyol –OH groups, again indicated by the FTIR band shifts.

A final point is the lower affinities of DPA phosphonate compared to the monoprotic phosphates (Figure 5) despite the presence of an additional strongly hydrogen bonding acid site, and the fact that phosphates are expected to have lower metal ion affinities compared to phosphonates due to the additional –O– on the phosphorus lowering the P=O basicity. This suggests that, while hydrogen bonding to the P=O enhances its affinity for metal ions, too much hydrogen bonding, as is the case with DPA, lowers the affinity by limiting access to the ligand. This concept was tested and successfully used in the development of a new fiber for the removal of uranium from seawater [25].

## 5. Conclusions

This report builds on earlier results with the phosphate diethyl ester polymers. Increasing hydrogen bonding through the presence of acidic –OH groups supplement that from any hydroxyl groups on a neighboring polyol. The increased bonding further increases the metal ion affinities of the monoprotic ligands and allows their use in solutions of much greater acidity than possible with the diethyl esters. Hydrogen bonding, thus provides another route to tuning metal ion affinities. The application of the concept to the recovery of uranium from highly acidic solutions of phosphoric acid, as well as from seawater will be reported.

## Figures and Tables

**Table 1:** Acid and phosphorus capacities of phosphonic acid and monoprotic polymers.

Resin	Acid Capacity (mmol/g)	Phosphorus Capacity (mmol/g)	Acid/Phosphorus
DPA	9.65	4.71	2.05
pEG1M	2.23	2.82	0.79
pGlyM	2.97	2.85	1.04
pPentaM	2.61	2.81	0.93

**Table 2:** Metal ion affinities of the monoprotic phosphates.

**pEG1M**				
**pH**	%Pb(II) abs (log D) *	**%Cd(II) abs (log D)**	**%Cu(II) abs (log D)**	**%Zn(II) abs (log D)**
1.41	95.5% (2.77)	83.1% (2.15)	82.7% (2.14)	62.2% (1.68)
1.00	80.9 (2.07)	54.7 (1.54)	53.6 (1.51)	23.6 (0.949)
0.699	47.7 (1.40)	36.6 (1.22)	24.6 (0.976)	10.8 (0.549)
0.398	19.5 (0.841)	11.8 (0.587)	8.15 (0.398)	0.990 (−0.545)
0.00	11.7 (0.567)	8.95 (0.448)	4.60 (0.145)	-
**pGlyM**				
**pH**	**%Pb(II) abs (log D)**	**%Cd(II) abs (log D)**	**%Cu(II) abs (log D)**	**%Zn(II) abs (log D)**
1.41	96.5 (2.91)	93.2 (2.60)	88.5 (2.35)	80.7 (2.08)
1.00	86.1 (2.25)	75.4 (1.96)	68.0 (1.79)	51.8 (1.49)
0.699	52.9 (1.53)	43.4 (1.35)	32.1 (1.15)	17.3 (0.775)
0.398	21.2 (0.893)	17.2 (0.786)	14.4 (0.683)	9.27 (0.483)
0.00	12.7 (0.638)	8.14 (0.418)	7.56 (0.389)	-
**pPentaM**				
**pH**	**%Pb(II) abs (log D)**	**%Cd(II) abs (log D)**	**%Cu(II) abs(log D)**	**%Zn(II) abs (log D)**
1.56	99.6 (3.84)	98.4 (3.26)	98.1 (3.17)	93.5 (2.63)
1.00	95.4 (2.79)	87.4 (2.30)	83.0 (2.16)	69.8 (1.82)
0.699	84.4 (2.21)	62.3 (1.68)	56.9 (1.60)	37.9 (1.24)
0.398	51.7 (1.51)	23.7 (0.955)	25.1 (1.01)	11.4 (0.562)
0.00	16.3 (0.762)	6.18 (0.292)	6.87 (0.332)	2.55 (−0.112)
**pEG1M**				
**pH**	**%Fe(III) abs (log D)**	**%Al(III) abs (log D)**	**%La(III) abs (log D)**	**%Lu(III) abs (log D)**
0.523	91.0 (2.46)	85.5 (2.22)	98.7 (3.33)	98.4 (3.17)
0.222	89.4 (2.37)	69.0 (1.79)	88.6 (2.34)	90.5 (2.43)
0.00	87.6 (2.32)	38.8 (1.27)	78.6 (1.99)	78.1 (2.00)
−0.301	80.1 (2.07)	14.9 (0.692)	46.7 (1.38)	56.1 (1.56)
−0.699	60.3 (1.62)	5.09 (0.183)	16.4 (0.708)	31.2 (1.10)
**pGlyM**				
**pH**	**%Fe(III) abs (log D)**	**%Al(III) abs (log D)**	**%La(III) abs (log D)**	**%Lu(III) abs (log D)**
0.523	99.1 (3.50)	98.6 (3.31)	99.6 (3.85)	99.9 (4.72)
0.222	98.7 (3.36)	92.6 (2.56)	95.3 (2.77)	97.9 (3.13)
0.00	98.5 (3.27)	76.0 (1.96)	90.1 (2.46)	91.7 (2.52)
−0.301	95.3 (1.77)	36.4 (1.23)	59.7 (1.67)	74.3 (1.93)
−0.699	89.3 (2.39)	15.5 (0.733)	23.7 (0.972)	49.5 (1.47)
**pPentaM**				
**pH**	**%Fe(III) abs (log D)**	**%Al(III) abs (log D)**	**%La(III) abs (log D)**	**%Lu(III) abs (log D)**
0.523	99.4 (3.71)	98.2 (3.21)	99.1 (3.53)	99.8 (4.11)
0.222	99.4 (3.71)	91.3 (2.49)	94.2 (2.67)	99.9 (3.63)
0.00	98.0 (3.15)	75.8 (1.97)	81.2 (2.12)	97.0 (2.96)
−0.301	97.5 (3.04)	43.8 (1.37)	59.1 (1.63)	87.8 (2.31)
−0.699	94.7 (2.72)	18.4 (0.821)	26.3 (1.00)	64.9 (1.75)

* log (distribution coefficient) = (mmol M*^n^*^+^_polymer_ per g_polymer_)/(mmol M*^n^*^+^_(final)soln_ per mL_(final)soln)_).

**Table 3:** Metal ion affinities of the diprotic phosphonic acid (DPA).

**pH**	**%Pb(II) abs (log D)**	**%Cd(II) abs (log D)**	**%Cu(II) abs (log D)**	**%Zn abs (II) (log D)**
1.64	94.3 (2.84)	86.8 (2.44)	83.4 (2.35)	78.6 (2.17)
1.00	60.1 (1.77)	34.5 (1.35)	28.2 (1.25)	21.4 (1.09)
0.699	26.6 (1.16)	9.58 (0.642)	11.5 (0.74)	12.8 (0.508)
0.398	10.4 (0.595)	5.05 (0.355)	2.48 (0.0658)	5.71 (0.412)
0.00	7.71 (0.535)	-	1.79 (−0.116)	-
**pH**	**%Fe(III) abs (log D)**	**%Al(III) abs (log D)**	**%La(III) abs (log D)**	**%Lu abs (III) (log D)**
1.00	99.6 (3.99)	98.8 (3.55)	98.1 (3.34)	99.8 (4.34)
0.523	-	91.2 (2.64)	68.3 (1.95)	95.2 (2.95)
0.222	98.6 (3.49)	62.0 (1.86)	23.5 (1.12)	80.7 (2.27)
0.00	97.2 (3.19)	33.8 (1.36)	11.1 (0.724)	52.2 (1.68)
−0.301	91.5 (2.67)	1.05 (0.271)	0.74 (−0.48)	24.5 (1.17)
−0.699	86.1 (2.41)	-	-	-
